# Pharmacokinetic properties and antitumor efficacy of the 5-fluorouracil loaded PEG-hydrogel

**DOI:** 10.1186/1471-2407-10-211

**Published:** 2010-05-18

**Authors:** Hee Yi, Hee-Jung Cho, Soo-Min Cho, Dong-Goo Lee, AM Abd El-Aty, So-Jeong Yoon, Gun-Won Bae, Kwang Nho, Bokyung Kim, Chi-Ho Lee, Jin-Suk Kim, Michael G Bartlett, Ho-Chul Shin

**Affiliations:** 1 Department of Veterinary Pharmacology and Toxicology, Konkuk University, Seoul 143-701, Republic of Korea; 2 SunBio Inc, Anyang 431-804, Republic of Korea; 3 Department of Physiology, School of Medicine, Konkuk University, Seoul 143-701, Republic of Korea; 4 Department of Food Science and Biotechnology of Animal Resources, Konkuk University, Seoul 143-701, Republic of Korea; 5 Department of Pharmaceutical and Biomedical Sciences, University of Georgia, Athens, GA 30602, USA; 6 Current address: Department of Pharmacology, Faculty of Veterinary Medicine, Cairo University, Giza, Egypt

## Abstract

**Background:**

We have studied the *in vitro *and *in vivo *utility of polyethylene glycol (PEG)-hydrogels for the development of an anticancer drug 5-fluorouracil (5-FU) delivery system.

**Methods:**

A 5-FU-loaded PEG-hydrogel was implanted subcutaneously to evaluate the drug retention time and the anticancer effect. For the pharmacokinetic study, two groups of male rats were administered either an aqueous solution of 5-FU (control group)/or a 5-FU-loaded PEG-hydrogel (treated group) at a dose of 100 mg/kg. For the pharmacodynamic study, a human non-small-cell lung adenocarcinoma (NSCLC) cell line, A549 was inoculated to male nude mice with a cell density of 3 × 10^6^. Once tumors start growing, the mice were injected with 5-FU/or 5-FU-loaded PEG-hydrogel once a week for 4 weeks. The growth of the tumors was monitored by measuring the tumor volume and calculating the tumor inhibition rate (IR) over the duration of the study.

**Results:**

In the pharmacokinetic study, the 5-FU-loaded PEG-hydrogel gave a mean residence time (MRT) of 8.0 h and the elimination half-life of 0.9 h; these values were 14- and 6-fold, respectively, longer than those for the free solution of 5-FU (p < 0.05). In the pharmacodynamic study, A549 tumor growth was significantly inhibited in the 5-FU-loaded PEG-hydrogel group in comparison to the untreated group beginning on Day 14 (p < 0.05-0.01). Moreover, the 5-FU-loaded PEG-hydrogel group had a significantly enhanced tumor IR (p < 0.05) compared to the free 5-FU drug treatment group.

**Conclusion:**

We suggest that 5-FU-loaded PEG-hydrogels could provide a useful tool for the development of an anticancer drug delivery system.

## Background

The drug 5-Fluorouracil (5-FU) is one of the most common chemotherapeutic agents used against malignant tumors [1]. However, this drug has some pharmacokinetic limitations including unfavorable maximum drug concentrations (Cmax) and short half lives following systemic bolus injection. Earlier reports have demonstrated that acute increases in plasma 5-FU concentration can cause severe side effects and the antitumor effects of 5-FU depend on exposure duration rather than plasma concentration levels [2]. Therefore, 5-FU acts more in a time-dependent manner than in a dose-dependent manner [3-5]. Therefore, a continuous infusion system for the maintenance of intended levels may be beneficial. However, this approach might be unfavorable due to its high cost and patient compliance with long-term regimens. Implantable release devices have been attempted in vivo to reduce the period of hospitalization and eliminate the need for indwelling catheters [6]. Recently, we have developed PEG-hydrogel derivatives as an injectable sustained release device which can be bolused subcutaneously without any surgical implantation (US Patent 6858736; Korean Patent KR 2002-0089772 and 10-2004-0040782). We have hypothesized that this approach can provide a diffusional barrier for drug release and thereby deliver drugs for an extended period of time. In the current study, we have evaluated the drug release profiles of a 5-FU-loaded PEG-hydrogel system. To confirm the therapeutic efficacy of the designed PEG controlled-release system, we conducted a pharmacodynamic study using the A549 tumor xenograft model in nude mice.

## Methods

### Chemicals and cell culture materials

The 6-arm PEG-SG (6-arm polyethylene glycol N-hydroxy succinimidyl glutarate), and 6-arm PEG-AM (6-arm polyethylene glycol amine) were developed by SunBio Inc. (An-yang, Republic of Korea). The 5-fluorouracil (5-FU), NaH_2_PO_4_, Na_2_HPO, methyl alcohol (analytical grade), and diethyl ether were supplied by Sigma (St. Louis, MO, USA). Dulbecco's modified Eagle medium (DMEM), penicillin/streptomycin, and trypsin EDTA were purchased from GIBCO (Carlsbad, CA, USA). Aqueous fetal bovine serum (FBS) was obtained from WelGENE (Daegu, Republic of Korea).

### Cell Line and Animals

For the pharmacokinetic study, male Sprague-Dawley rats were obtained from Orient Bio Inc. (Seongnam, Republic of Korea). The A549 human lung carcinoma cell line, a generous gift from Cha Biomedical Center (Seoul, Republic of Korea), was carefully inoculated into the dorsal neck of male nude mice (Balb/c Slc-nu/nu), which were supplied from the Central Lab Animal Inc. (Seoul, Republic of Korea). All *in vivo *experiments were carried out with the approval of the *Institutional Animal Care and Use Committee *(IACUC) at Konkuk University and in harmony with *the Guide for Laboratory Animal Care and Use (*IACUC Approval No. KUV7015, 7016)

### Preparation of 5-FU-loaded PEG hydrogel and in vitro Release of 5-FU-loaded PEG-hydrogel

**Figure 1:**
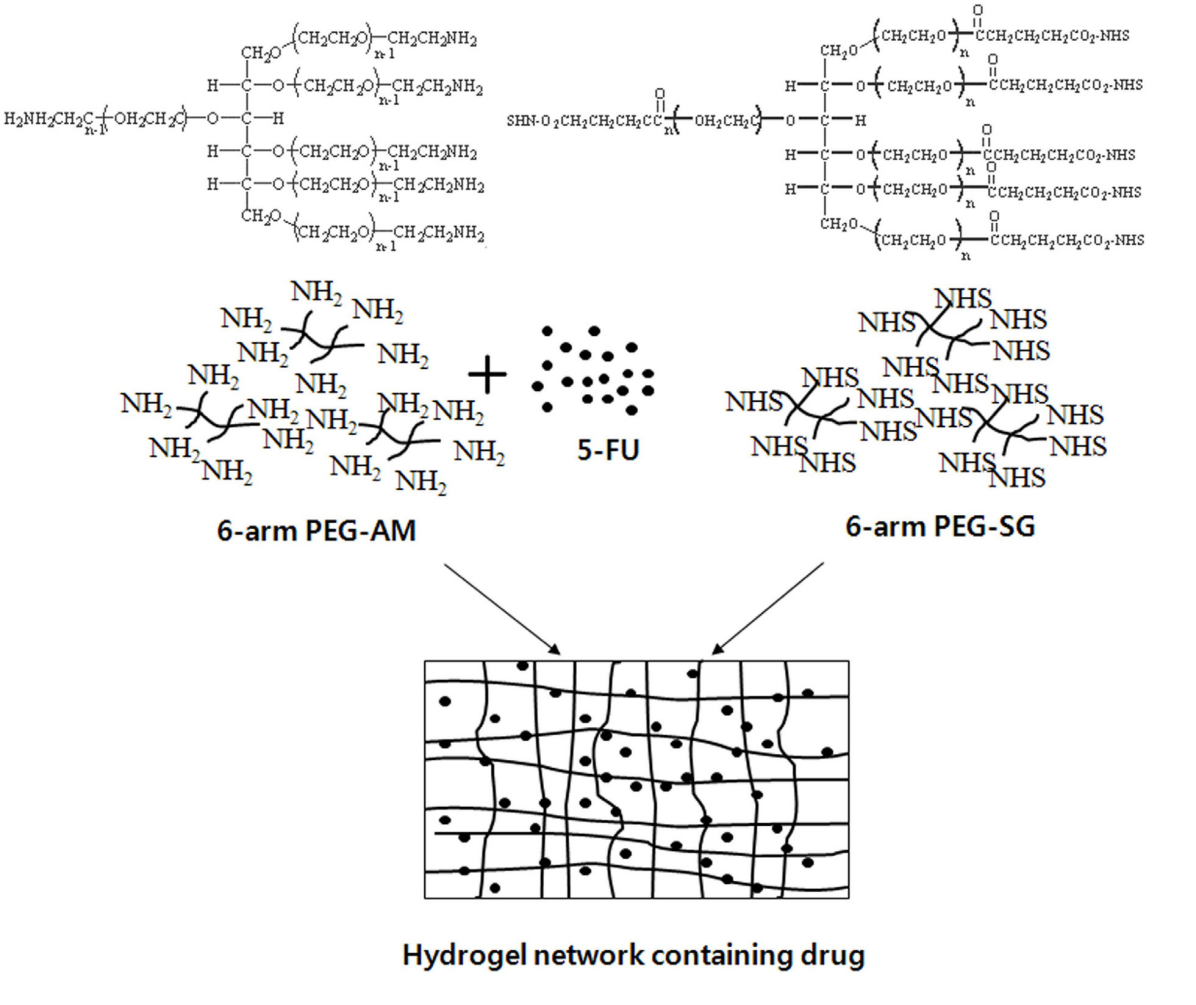
**The chemical structures of the 6-arm PEG- AM and 6-arm PEG- SG**. PEG-hydrogel network made by 6-arm PEG-AM and 6-arm PEG-SG. After displacement of the NHS (N-hydroxy succinimide) from 6-arm PEG-SG, amine residues on 6-arm PEG-AM are cross-linked with 6-arm PEG-SG.

The 5-FU-loaded PEG-hydrogel was designed as shown in Fig 1. The 6-arm PEG-AM was dissolved in 10 mM phosphate buffer (pH 6.0), and 5-FU was added into the solution at a concentration of 1 mg/ml. The 6-arm PEG-SG was also dissolved in 10 mM phosphate buffer (pH 6.0). The 6-arm PEG-AM and 6-arm PEG-SG solutions were prepared at various concentrations (i.e., 7, 10, and 15% w/v). Approximately, 0.5 ml of 6-arm PEG-AM solution containing 5-FU was placed in a 6-well plate, and an equal volume of 6-arm PEG-SG solution (0.5 ml) was added and mixed. The plate was shaken vigorously until the solutions hardened, forming the hydrogel. After hardening, the hydrogel was washed gently with 1 ml of 10 mM phosphate buffer (pH 7.2) to remove the any residual 5-FU left on the hydrogel surface. The *in vitro *release study was carried out over 8 days. To quantify 5-FU released from the hydrogel, 2.5 ml of 10 mM phosphate buffer (pH 7.2) was added into each well and replaced every day. The plate was sealed and placed at room temperature. The quantity of 5-FU released from the hydrogel was measured by monitoring the absorbance at 265 nm. Since the released NHS or other PEG compounds gave additional signals at this wavelength, we carried out an additional experiment to assess background values without 5-FU. We calculated the net concentration of 5-FU by subtracting the background signals resulting from other compounds from the total signal.

Fig. 1 shows the cross-linking reaction of 6-arm PEG-amine (PEG-AM) and 6-arm PEG-succinimidyl glutarate (PEG-SG). When the PEG-SG is mixed with PEG-AM, PEG-SG separates the N-hydroxy succinimides (NHS) from its arms. Additionally, the terminals of PEG-SG (-CO_2_) are cross-linked with the amine (-NH_2_) groups of PEG-AM. This cross-linking between PEG-AM and PEG-SG changes the two PEG solutions into a gelatinous form (US Patent 6858736).

### Pharmacokinetics

Rats were divided into two groups: a free 5-FU treatment group and a 5-FU-loaded PEG-hydrogel group. An aqueous solution of free 5-FU and a 5-FU-loaded PEG-hydrogel were administered subcutaneously to the rats at a dose of 100 mg/kg. To formulate the 5-FU-loaded PEG-hydrogel, PEG-SG and PEG-AM containing 5-FU were dissolved in sodium phosphate buffer (pH 8.0) and mixed together in equal volumes. The free 5-FU drug solution was injected into rats using a normal syringe, and the 5-FU-loaded PEG-hydrogel was injected using a mixing syringe device (Doowon Meditec Corp., Youngin-city, Republic of Korea). The aqueous solution of PEG-hydrogel (PEG-SG and PEG-AM) immediately changed into a gel after passing through the injection needle. Blood samples were collected at minutes (0, 5 and 30) and hours (1, 2, 3, 4, 12, and 24) after drug administration. Sampling continued until the PEG-hydrogel could not be palpated under the skin. Blood samples were centrifuged at 8000 rpm for 5 minutes (HANIL Science Industrial Co., Inchon, Republic of Korea), and harvested serum (150-200 μl) was subjected to HPLC analysis [7]. Samples were extracted using ethyl acetate (6~8 ml) and then evaporated to dryness under N_2 _gas in a water bath adjusted at 60°C. A 1.5 mM sodium phosphate buffer (pH 5.8) was added to reconstitute the residue. Approximately 200-300 μl of the reconstituted solutions were filtered and injected for HPLC (Agilent 1100, Santa Clara, CA, USA). Separation was accomplished via isocratic elution of the mobile phase, which contained methanol and 1.5 mM sodium phosphate buffer (99:1, v/v, pH 5.8), with a flow rate of 1 ml/min. A C_18_(Capcell Pak UG120, 5 μm, 4.6 μm I.D. × 250 μm, Shiseido, Tokyo, Japan) was used as an analytical column. The analysis was carried out at a column temperature of 40°C. The wavelengths of the FLD (fluorescence detector) were 260 nm and 350 nm for excitation and emission, respectively.

Pharmacokinetic parameters were estimated for each rat by using the program "*WinNonlin*" to fit the serum concentration *versus *time data to the following equation: *C*_*p *_= (*K*_a_*F*D)/{V_d_*(k_*a*_-*k*_*el*_)}*(exp^-kel*t ^- *exp*^-ka*t^), where *C*_*p *_is the serum concentration and k_a _and k_el _are the absorption and the elimination rate constants, respectively. The F, D, and V_d _represent the bioavailability, dose, and volume of distribution, respectively. The lag time was not considered, and the absorption and elimination rates were applied using first-order kinetics. The area under the concentration *vursus *time curve (AUC^0-8 day^) was calculated using the trapezoidal rule from time *t *= 0 to the last measured concentration on Day 8. Serum drug concentrations and the estimated pharmacokinetic parameters were reported as means ± SD.

### Antitumor Activity of 5-FU-loaded PEG-hydrogels in Tumor-bearing Nude Mice

A total of 3 × 10^6 ^of cells from the A549 line, a human non-small-cell lung adenocarcinoma cell line, were inoculated into the dorsal neck of 4-week-old male nude mice (Balb/c Slc-nu/nu) as described in earlier reports [7,8]. The tumor mass was monitored every week. When the tumors grew to 100-400 mm^3^, the mice were randomized into three groups: an untreated control group, a free 5-FU drug control group, and a 5-FU-loaded PEG-hydrogel group. The free 5-FU drug and 5-FU-loaded PEG-hydrogel were prepared as described above was subcutaneously injecting around the tumor mass once per week for 4 weeks at a dose of 85 mg/kg. Body weight, tumor volume, and clinical assessment of the mice were monitored every week until the end of the study. There was no significant difference in body weight changes among the groups. When tumors were palpable and visible, the tumor volume (TV) was measured with a Vernier caliper and calculated using the following formula:

The antitumor effect was estimated by calculating the relative tumor volume and the relative tumor inhibition rate (IR, %). The relative tumor volume (RTV) represents the tumor volume when the drugs are given to the mice.

where V_0 _is the tumor volume when the drugs are given to the mice and V_t _is the tumor volume at each measurement.

The T_RTV _represents the RTV of treated groups, and the C_RTV _represents the RTV of the untreated control group.

### Histopathological Examination

Sections of A549 taken from subcutaneously transplanted tumor masses were fixed with formalin and embedded in paraffin. Five micrometer tissue sections were prepared and stained with H & E.

### Statistical Analysis

Multiple comparison tests for different treatment groups were conducted. An ANOVA multiple comparison test (Dunnett's test) was performed to determine which pairs of groups differed significantly. An unpaired Student's *t*-test was also used to compare the 5-FU-loaded PEG-hydrogel group *versus *the 5-FU-treated control group. The level of significance was taken as p < 0.05 or 0.01. Values represent means ± SD. Statistical analyses were performed with Statistical Analysis Systems software (SAS/STAT Version 8.1, Cary, NC, USA).

## Results

### *In vitro *Release of 5-FU from PEG-hydrogel

**Figure 2:**
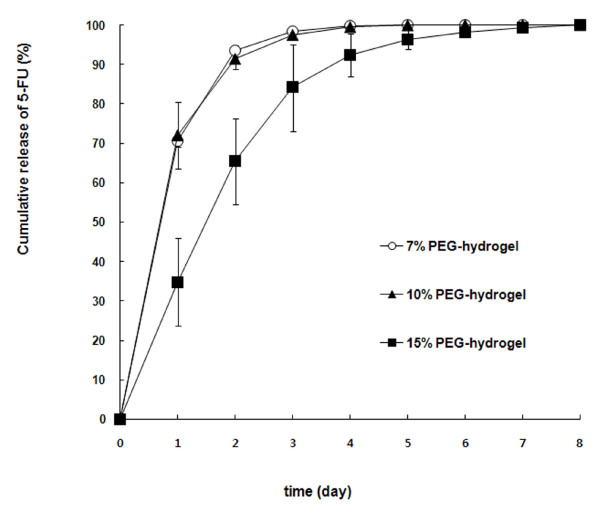
***In vitro *release of 5-FU from PEG-hydrogel**. The error bars represent the range from two experiments.

The optimal cross-linking reaction was obtained using 1 mM phosphate buffer (pH of 8.0). Fig. 2 presents the cumulative releases of 5-FU from the 5-FU-loaded PEG-hydrogel at 7, 10, and 15% PEG concentrations. The 15% PEG-hydrogel released 5-FU much more slowly than did the 7% and 10% PEG-hydrogels. Most of the 5-FU in the 7% and 10% of PEG-hydrogels was released within 2 days, whereas the 15% PEG-hydrogel released 5-FU over a period of 5 days.

### Pharmacokinetics of the Free 5-FU Drug and 5-FU-loadedPEG-hydrogel

**Figure 3:**
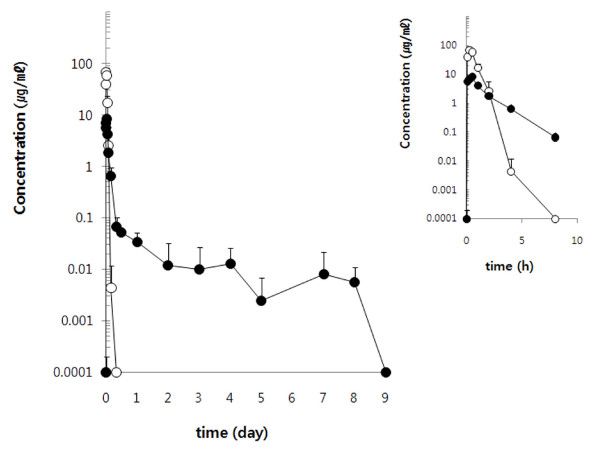
**Serum concentration curves for 5-FU following subcutaneous injection of 100 mg/kg of the free 5-FU drug (open circle) and 5-FU-loaded PEG-hydrogel (closed circle) to SD rats (n = 3)**. Right panel shows serum concentration curves through 0 to 8 hour.

Fig. 3 shows the average serum concentration *versus *time curves of 5-FU following subcutaneous administration of both the free 5-FU drug and 5-FU-loaded PEG-hydrogel to rats at 100 mg/kg. The 5-FU-loaded PEG-hydrogel released 5-FU into the serum over a period of one week, whereas the 5-FU from the free drug injection rapidly disappeared from the serum within several hours after injection. The serum concentration profiles for the free 5-FU and 5-FU-loaded PEG-hydrogel were well described by a one-compartment open pharmacokinetic model. As shown in Table 1, between the two groups there were marked differences in some parameters including the maximum serum concentrations (C_max_), the elimination half-lives (t_1/2_) and the area under the curves (AUC). The C_max _and t_1/2 _in the free 5-FU treated group were about 68 μg/ml and 0.15 h, respectively, while those parameters in 5-FU-loaded PEG-hydrogel group were 8 μg/ml and 0.9 h, respectively. In the free 5-FU treated group, the AUC and the area under the moment curve (AUMC) were roughly 60 μg h/ml and 33 μg h^2^/ml, respectively. In the 5-FU-loaded PEG-hydrogel group, the AUC and AUMC were 14 μg hr/ml and 112 μg h^2^/ml, respectively.

**Table 1:** Pharmacokinetic parameters of 5-FU after the subcutaneous injection of 100 mg/kg of the free 5-FU drug and 5-FU-loaded PEG-hydrogel into SD rats.

Parameters	unit	5-FU (n = 3, Mean ± SD)	5-FU-loaded PEG-hydrogel (n = 3, Mean ± SD)
V_d_/F	L	0.41 ± 0.04	10.60 ± 1.55
t_1/2(a)_	h	0.21 ± 0.06	0.06 ± 0.03
t_1/2_	h	0.15 ± 0.04	0.87 ± 0.17*
CL_tot_/F	l/h	2.04 ± 0.51	8.46 ± 1.07
C_max_	μg/ml	67.81 ± 15.84	7.47 ± 1.63
T_max_	h	0.25 ± 0.00	0.33 ± 0.14
AUC^0-8 day^	μg·h/ml	59.71 ± 10.58	14.14 ± 1.20
MRT	h	0.55 ± 0.03	8.03 ± 4.41*

V_d_/F; apparent volume of distribution, t_1/2(a)_; absorption half life, t_1/2_; elimination half life, CL_tot_/F; total body clearance, C_max_; maximum serum concentration, T_max_; time to maximum concentration, AUC; area under the curve, MRT; mean residence time (AUMC/AUC). * 5-FU *vs*. 5-FU-loaded PEG-hydrogel (p < 0.05).

### Tumorigenicity of the A549 Xenograft and Histopathology

**Figure 4:**
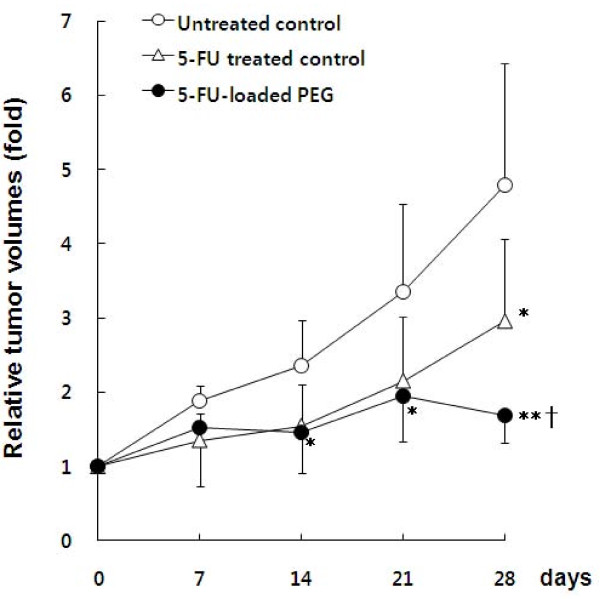
**Comparison of relative tumor volumes among treatment groups**. * Compared to the untreated control group (n = 6, * p < 0.05, ** p < 0.01). ^† ^Compared between 5-FU-treated control and 5-FU-loaded PEG-hydrogel groups (n = 6, † p < 0.05).

A total of 3 × 10^6 ^A549 cells were inoculated into nude mice. The subcutaneous inoculation of tumor cells resulted in tumor generation at the injection site. Solid tumors were locally measurable by 1 month after inoculation. As shown in Fig. 4, the relative tumor volume (RTV) of the untreated control group markedly increased in a time-dependent manner. However, the RTVs were significantly suppressed compared to the untreated group by 5-FU-loaded PEG-hydrogel treatment starting on Day 14. However, the free 5-FU drug also showed significant inhibition of tumor volume increases on Day 28. The histopathological examination revealed typical adenocarcinoma patterns, including the mixed acinar and tubular form characterized by acini and tubules composed of cuboidal and columnar cells. Poorly differentiated tumor cells are shown among the cells. Some cancer cells infiltrated into the muscular layer.

### Comparative Antitumor Effects of the Free 5-FU and 5-FU loaded PEG-hydrogel

Compared to the free 5-FU-treated group, A549 tumor growth was significantly inhibited in the 5-FU-loaded PEG-hydrogel group on Day 28 (p < 0.05). The 5-FU-loaded PEG-hydrogel produced an inhibition rate of 64%, and the free 5-FU drug produced an inhibition rate of 38%. The PEG-hydrogel itself showed no cytotoxicity for A549 cells and had no inhibitory effect on tumor growth in the mouse xenograft (data not shown).

## Discussion

Because the use of 5-FU is limited by its short half-life and rapid elimination, short duration 5-FU bolus administration has a relatively low response rate [9]. For these reasons, suitable methods for the administration of 5-FU have been investigated for a long time [10-12]. Since continuous intravenous infusion has the advantage of maintaining a low concentration of the drug in the blood for an extended period of time, continuous 5-FU infusion has been more effective than bolus administration in clinical trials [13,14]. In comparison to bolus administration, continuous infusion also minimizes the severe, life-threatening toxic effects of 5-FU [15,16]. Accordingly, continuous infusion is an effective method for both increasing the length of time for which the drug contacts tumor cells and reducing toxicity. However, continuous infusion may cause patient discomfort due to indwelling catheters and bed restriction, and it may give rise to complications like catheter-related vasculitis and sepsis [15,17]. Therefore, controlled-release devices (e.g., biodegradable polymers) have been previously studied for the development of anticancer drug delivery [18,19].

An optimal crosslinking reaction of PEG-SG and PEG-AM was observed in 1 mM phosphate buffer at a pH of 8.0. PEG-hydrogel formation was maximized by using 15% concentrations of PEG-AM and PEG-SG. These conditions of PEG-hydrogel formation were applied in our *in vivo *pharmacokinetics and antitumor screening studies. Furthermore, this PEG-hydrogel system is based on a very simple injection that does not require a surgical procedure.

Many other groups have reported that the pharmacokinetics of 5-FU in patients vary significantly based on the dosing regimen [20]. In addition, like other anticancer chemotherapeutics, 5-FU has a relatively narrow therapeutic index [21]. For these reasons, controlling the serum concentration of the drug is important for drug efficacy and safety. When injected subcutaneously in our study, 5-FU disappeared rapidly from systemic circulation. On the other hand, the elimination of 5-FU from the 5-FU-loaded PEG-hydrogel showed significantly delayed release from systemic circulation. The mean residence time of 8 hours is approximately 14 times higher than that of free 5-FU administered to animals. Moreover, the elimination half-life of the 5-FU-loaded PEG-hydrogel was 0.9 h; this is 6-fold larger than that of the free 5-FU-treated control group. Blanco et al. [22] also evaluated the drug release profile of a subcutaneously implanted poly (2-hydroxyethylmethacrylate-co-acrylamide) hydrogel containing 5-FU. In this study, the drug was released from the poly hydrogel over a span of 2 days. In our study, the PEG-hydrogel released 5-FU more than 4 days after the injection. Although Blanco et al. [22] employed a surgical incision for the implantation of the poly hydrogel, we implanted the hydrogel by direct injection using a mixing syringe device in which the SG and AM solutions were immediately changed into a solid gel by mixing together.

We report that the controlled release of 5-FU from by the PEG-hydrogel effectively suppressed tumor growth *in vivo*. Compared to the free 5-FU-treated control group, the 5-FU-loaded PEG-hydrogel group demonstrated a strong tumor growth inhibition effect. Following injection once a week, the tumor inhibition rate (IR) of the 5-FU-loaded PEG-hydrogel animals markedly increased from 20% (Day 7) to 65% (Day 28). In contrast, the IR of animals in the free 5-FU-treated control group showed a smaller increase from 30 to 40%. These pharmacodynamic data demonstrate that the PEG-hydrogel system is very effective in maintaining the optimal blood concentration of 5-FU necessary to suppress growth of tumor cells efficiently. The current finding is in agreement with the fact that 5-FU is not a dose-dependent but rather a time-dependent drug [3-5].

The common acute toxic effects of 5-FU include myelosuppression, mucositis, and diarrhea [15,23,24]. It is generally understood that the toxicity of 5-FU is related to the AUC [20,25,26]. In our pharmacokinetic studies, the AUC and C_max _of 5-FU were markedly decreased after treatment with the PEG-hydrogel system. These findings may suggest that 5-FU treatment with a PEG-hydrogel may be used to reduce the severe toxic effects of this drug.

## Conclusions

Our results suggest that the injectable PEG-hydrogel system offers an efficient approach to cancer therapy using a direct injection method that circumvents surgical incision.

## Abbreviations

(PEG): polyethylene glycol; (5-FU): 5-fluorouracil; (IR): inhibition rate; (MRT): mean residence time; (PEG-SG,): polyethylene glycol N-hydroxy succinimidyl glutarate; (PEG-AM): polyethylene glycol amine; (TV): tumor volume; (RTV): relative tumor volume; (C_max_): maximum serum concentration; (T_max_): time to maximum concentration; (t_1/2_): elimination half-life; (AUC): area under the curve; (AUMC): area under the moment curve.

## Competing interests

The authors declare that they have no competing interests.

## Authors' contributions

HY, KN and HS conceived the study and finalized the manuscript. HY, HC, SC and DL carried out the sample preparation, pharmacokinetics, histopathological examination and *in vivo *and *vitro *antitumor studies. SY, GB and KN developed the 6-arm PEG-SG and 6-arm PEG-AM, and performed *in vitro *release study of 5-FU from PEG-hydrogel. AMAE, BK, CL, MGB and JK contributed to the scientific discussion and the manuscript editing. All authors read and approved the final manuscript.

## Pre-publication history

The pre-publication history for this paper can be accessed here:

http://www.biomedcentral.com/1471-2407/10/211/prepub
